# Addition of Modified Lemaire Lateral Extra-Articular Tenodesis in a Single stage Revision Anterior Cruciate Ligament Reconstruction using Peroneus Longus Tendon: A Prospective Study

**DOI:** 10.5704/MOJ.2503.004

**Published:** 2025-03

**Authors:** SS Sonarkar, A Stanley, S Kumar-Singh, R Garg, A Narula, M Raj

**Affiliations:** 1 Department of Orthopaedics and Rehabilitation, 7 Air Force Hospital, Kanpur, India; 2 Department of Orthopaedics, Maa Vindhyavasini Autonomous State Medical College, Mirzapur, India; 3 Department of Orthopaedics, Ganesh Shankar Vidyarthi Memorial Medical College, Kanpur, India; 4 Department of Orthopaedics, Dr Aditya Narula, Aakaar Bone Care, Kanpur, India; 5 Department of Orthopaedics, All India Institute of Medical Sciences, Deoghar, India

**Keywords:** lateral tenodesis, revision anterior cruciate ligament, return to activity, graft failure, Lemaire

## Abstract

**Introduction:**

The purpose of this study is to assess the outcomes of patients that underwent single-stage revision ACL reconstruction (ACLR) with peroneus longus tendon (PLT), augmented with lateral extra-articular tenodesis (LET) using the modified Lemaire technique.

**Material and Methods:**

All the 18 patients underwent arthroscopic single-stage revision ACLR using autologous PLT with an additional modified Limier LET procedure. Patients were thoroughly assessed pre- and post-operatively by the Lachman test, the pivot shift test, and the side-to-side difference by the Rolimeter. Functional evaluation was done with the help of the Lysholm score, the IKDC subjective score, Tegner score, VAS score, MARX activity rating scale and The American Orthopaedic Foot and Ankle Society (AOFAS) score. Post-operatively, patient satisfaction, return to sport, and physical activity were also recorded. SPSS ver. 22.0 software was used. Wilcoxon test, paired and unpaired t-tests were used to compare variables. Statistical significance was determined by a two-sided p-value <0.05.

**Results:**

Regarding subjective evaluations; post-operative residual laxity, and return to sport and physical activity, all of the patients demonstrated excellent results. Post-operatively, there was significant improvement in the anterior knee laxity. According to the Marx Activity Rating Scale, the extent of sports engagement was significantly increased at 18 months following surgery (p<0.001). According to the AOFAS score (p=0.38), there were no documented significant donor site morbidities.

**Conclusion:**

Single-stage revision ACLR using PLT with an additional modified Lemaire LET procedure results in a significant reduction in residual knee laxity with good clinical outcomes and a high return to play and physical activity.

## Introduction

The prevalence of revisions due to primary surgical failure is rising along with the number of primary ACLR are being performed^1,2^. Moreover, it has been determined that peripheral knee structures and the knee's bony alignment, such as lesions of the anterolateral structures or an elevated posterior tibial slope (PTS), are risk factors for the failure of ACLR^3^. The primary issue following intra-articular ACLR is the continuation of pivot shift, which increases the chance of revision ACLR^4,5^. In fact, after an isolated ACL restoration, 25% to 38% of patients still experience a pivot shift^6^.

Many biomechanical research studies have shown that anterolateral ligament (ALL) insufficiency causes high-grade anterior laxity of the knee and increases anterior knee translation, pivot shift, and internal rotation^7^. While research has shown that high-grade anterior laxity of the knee is a potential risk for ACLR failure, LET, which is becoming more popular after the rediscovery of the ALL by Claes *et al* in 2013, also lowers the likelihood of ACLR and revision ACLR failure^8,9^. An LET improves patient-reported and radiographic results, decreases residual anterolateral rotational instability, as well as the re-rupture rate after ACLR^9,10^. A LET also limits laxity and excessive internal rotation^11,12^.

The evidence states that higher grade pivot-shift, gross ligamentous laxity, genu recurvatum, and young patients returning to high level contact sports are indications for an additional LET^13,14^. However, long-term development of lateral tibiofemoral osteoarthritis as well as joint stiffness and loss of range of motion in flexion and extension are potential side effects of LET procedures; some research has challenged these outcomes, claiming that LET with ACLR lowers the risk of osteoarthritis and subsequent meniscal lesions^3,15-17^.

The management of rotatory instability has been recommended using a number of extra-articular techniques. The literature has covered the MacIntosh, Lemaire, and ALL-reconstruction procedures^14,18-20^. It has been demonstrated that the modified Lemaire approach reduces the pivot shift test while having a low complication rate^21^. Biomechanical evidence suggests that a modified Lemaire tenodesis remains equally effective as ALL reconstruction^22^.

Therefore, it is reasonable to suppose that, in the presence of an ACL injury and lesion of the anterolateral structures, a modified Lemaire tenodesis can more accurately restore native rotational knee kinematics than the ALL approach^22^.

The hamstring tendons (semitendinosus and gracilis), bone-patellar tendon-bone (BPTB), and quadriceps tendon are the most frequently used autografts. The hamstring tendons have become the preferred graft because they are relatively easier to harvest, have less donor site morbidity, and have a tensile strength similar to that of the native ACL^4,17^. However, its drawbacks include variable graft size, the potential for saphenous nerve damage, and a potential decline in the strength of hamstring muscle that could cause a quadriceps-hamstring imbalance, which is essential for some athletes who require dominant hamstring power^17,23^. PLT for routine ACLR has recently been explored as a possible graft of preference^23,24^. Additionally, there is no chance of hamstring muscles weakening following surgery or saphenous nerve harm during graft retrieval, and it has good biomechanical characteristics and a significant load-to-failure resistance^25^. There is, however, a lack of data on the outcomes of combining LET with revision ACLR using PLT autograft in adult-age patients with pre-operative high-grade anterior instability of the knee who engage in high-level contact activity.

The purpose of this study was to assess clinical outcomes, complications, and rate of return to pre-injury physical activity and sport level in adult patients who underwent combined single-stage ACLR with LET using autologous PLT graft, at a minimum of 18 months of follow-up.

## Materials and Methods

**Fig. 1:**
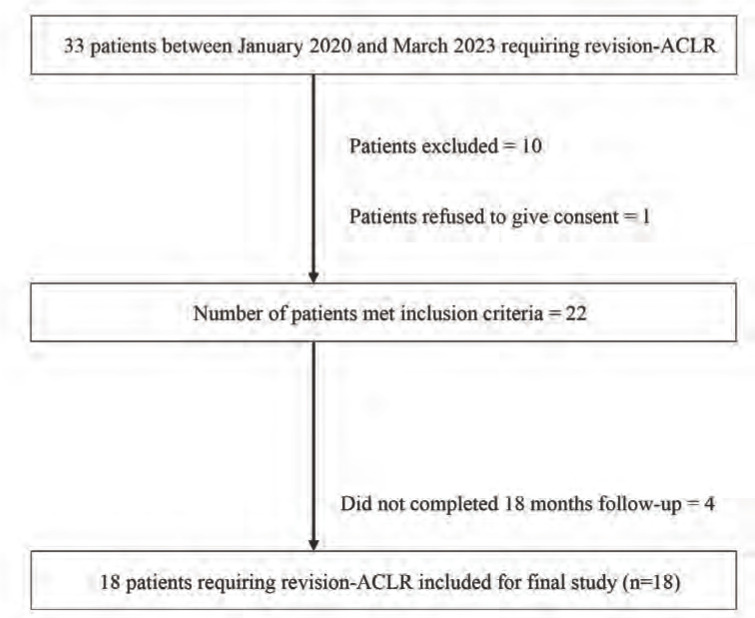
A flowchart showing the patient enrolment and the final study sample size.

The aim of this prospective study was to access the outcomes of a single-stage revision ACLR using autologous PLT graft in which modified Lemaire lateral extra-articular tenodesis was additionally utilised, with a minimum follow-up of 18 months. Thirty patients with primary ACLR were admitted to the Department of Orthopaedics at a tertiary care centre in the northern part of Uttar Pradesh, India, between January 2020 and March 2023 due to persistent or recurrent knee instability. Of these, 22 patients diagnosed with failed ACLR and who met the study's inclusion criteria were enrolled as study participants. All of these patients underwent with single-stage revision ACLR with modified Lemaire LET using ipsilateral autologous PLT graft. Four patients were not found throughout the minimum 18-month follow-up period required, and as a result, they were excluded from the study. The final study population had a total of 18 participants. Fig. 1 shows the flow chart that illustrates how patients were enrolled in the present study.

Prior to the start of the study, clearance had been obtained by the Institutional Ethical Committee (IEC SI No. 7AFH/2794/1B/Med Trg). A written informed consent was obtained from all the participants before they were enrolled in the study. Each author has reviewed the manuscript and agrees that it accurately represents the collective effort of everyone involved.

Inclusion criteria included skeletally mature patients above the 20-years age group, persistent or recurrent instability of the knee joint after primary ACLR, high-grade anterior knee laxity (grade 3 pivot shift, examination under anaesthesia) at the time of revision surgery, side-to-side differences (SSD) of more than six millimetres by the testing with Rolimeter®, and the date of re-injury to the date of surgery between 12 weeks and 12 months. The patients were excluded if they presented with multiligament injury of knee joint, patients requiring two-stage revision ACLR due to deficiency in bony tunnels, generalised joint hypermobility (Beighton score ≥ 4/9)^26^, posterior tibial slop (PTS) greater than 120, infection, BMI > 35 kg/m^2^, refused to give consent, or had a follow-up of less than 18 months. The Lysholm score^27^ and Tegner scale^28^ were calculated prior to the revision ACLR, and a thorough clinical pre-operative physical examination was performed when the patient was under anaesthesia. Standing radiographs of the entire leg were taken to identify any leg malalignment. PTS was measured as the angle between the perpendicular of the anatomic axis of the lateral tibia and the tangent over the medial tibial plateau. A pre-operative 3D-CT scan was also taken to assess the position and diameter of the tunnels. A pre-operative MRI was also obtained to assess the condition of the primary ACL graft, other ligaments, and soft tissues of the knee joint.

The pivot-shift test was classified into absent, Grade 1 (glide), Grade 2 (clunk), and Grade 3 (subluxation) while the Lachman test was performed using the 2000 Knee Examination Form from the International Knee Documentation Committee (IKDC) (absent, Grade 1: two to five millimetres, Grade 2: six to 10mm, and Grade 3: >10mm)^29^.

Our graft of choice for revision ACLR was the PLT autograft harvested from the ipsilateral leg. All the surgeries were done in supine position under spinal anaesthesia with pneumatic tourniquet cuffed at the upper thigh. Under anaesthesia, Lachman and pivot-shift tests were performed to confirm ACL insufficiency, along with the examination of the medial collateral ligament (MCL), the lateral collateral ligament (LCL), and the posterior cruciate ligament (PCL) for stability of these ligaments.

A thorough diagnostic arthroscopy assessment was done. A meniscal tear was repaired, or a partial meniscectomy was done in case of grossly irreparable meniscal tears. The residual graft was debrided. If the notch's width was less than 15mm, the orientation of the tibial and femoral tunnels inside the joint was evaluated, and a notchplasty was carried out. At this time, any femoral interference screws that would have prevented the drilling of the revision tunnel were removed.

Keeping the identical intraarticular position and pivoting the tibial aimer to a medial spot on the tibia, halfway between the tubercle and the posteromedial border, allowed grafts in a vertical position to be revised with an appropriate insertion site. We positioned the intra-articular tibial tube in the native footprint along the medial tibial spine downslope at the posterior border of the anterior horn lateral meniscus. This was 8mm anterior to the PCL's tibial insertion. The femoral tunnel was best for the right and left knees at 10-10:30 or 1:30-2 on the clock face.

We lifted the tibial aimer parallel to the plateau in the sagittal plane. The guide wire has been inserted and the tibial tunnel reamed. Sequential reaming can align tunnels if a preceding tunnel or interference screw precludes the use of a larger reamer. The tibial tunnel was used to implant the femoral over-the-top guide after removing soft tissue and bone debris from the knee.

Pre-operatively identifying tunnel widening and planning for tunnel management is crucial for revision procedures. If the primary screws were replaced with biocomposite screws earlier in the case and the screw was noticed during reaming, pre-tap the track for a new interference screw. For the substantial bone void, stacked biocomposite screws were employed. For graft harvesting, the ipsilateral side of the limb was used by keeping the foot in inversion, and a 4-5cm long incision was made along the long axis of the leg, posterior to the lateral malleolus. Layer by layer dissection was done, and the peroneus longus and another tendon with the muscle belly, the peroneus brevis, were identified. A surgical knot was tied around both tendon stumps. The peroneus longus stump was cut at 1cm proximal to the knot and whipstitched. A closed tendon stripper was used to harvest the graft proximally, up to five fingers breadth below the fibular head.

Once we are finished with revision ACL reconstruction surgery, we move for LET by modified Lemaire procedure. The knee was flexed to 900 and marking was done by identifying the fibular head moving up laterally in line with the iliotibial band direction. An 8–10cm long incision was made over the lateral epicondyle extending proximally. The iliotibial band was identified, and a strip of 10mm width and 10cm long distal tractus-iliotibialis attached with Gerdy tubercle was dissected, taking care not to damage the lateral collateral ligament (LCL).

**Fig. 2:**
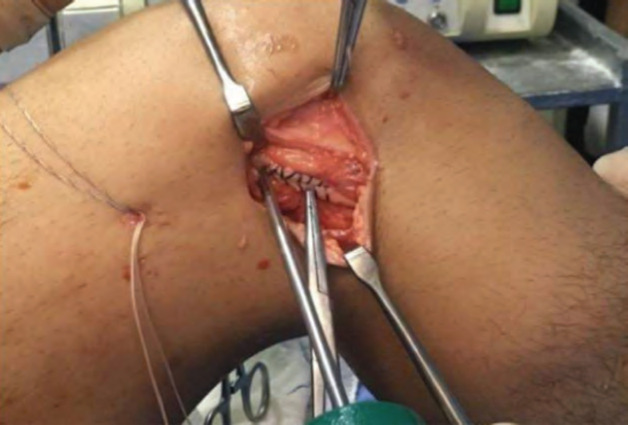
Figure showing the surgical technique of lateral extra-articular tenodesis. Around 10cm long IT band stripped off and Krackow sutures taken with high tensile suture material and shuttled under LCL.

Using a high-tensile suture material, Krackow whipstitching sutures were inserted as per standard technique and shuttled beneath the LCL (Fig. 2). Upon determining the interval between the gastrocnemius tubercle and the lateral epicondyle, a guide wire was drilled at a 30° anterior and proximal angle. A 7-mm drill is then used to over-drill the guide wire in neutral foot rotation and 30° of knee flexion. The ITB strip was shuttled into the drillhole and secured with an interference screw of the same diameter. Post fixation stability of the knee was assessed on the table.

Post-operative follow-up was done at 14 days, 1 month, 3 months, 6 months, 12 months, and 18 months after the surgery. At the time of the follow-up, along with the clinical assessment, the Lysholm, Tegner, and subjective International Knee Documentation Committee (IKDC) scores were also recorded^30^. Additionally, the visual analogue scale (VAS) was used to assess the individual's level of apprehension^31^. A self-reported dichotomous questionnaire was administered pre-operatively and post-operatively at 18 months to evaluate the patient's return to sport and physical activity, as well as the patient's level of satisfaction with the surgical outcome. The MARX-activity scale, a four-item activity assessment scale that has been demonstrated to have the greatest reliability in patients who have knee injuries, was used to measure patients' ability to return to their previous level of activity^32^. The participants will be given a scale from 0 to 4 on which to record the number of times they were able to run, cut, decelerate, and pivot in their most active phase throughout the course of the previous year. The highest possible score is sixteen points. At the eighteen-month follow-up, the American Orthopaedic Foot and Ankle Society (AOFAS) score was used to evaluate the ankle morbidity on the donor side^33^. Post-operative failure of revision ACLR was determined to have occurred when the Rolimeter test revealed a variation of 5 ≥ millimetres from side to side, in addition to a pivot-shift grade of 2 or 3.

Continuous data is represented as the mean and standard deviation, while categorical variables are shown as numbers and percentages. When necessary, the Wilcoxon test was used to compare continuous and ordinal variables, while paired and unpaired t-tests were used to compare continuous variables. Statistical significance was set at a two-sided p-value <0.05.

## Results

A single-stage revision ACLR procedure was carried out on 18 patients using an autologous PLT-graft and LET (modified Lemaire) technique. Table I summarises the demographic characteristics, details of primary surgery and intra-operative findings. All the patients were male with mean age of 30.1±11.8 years. Cartilaginous lesion was present in 77.78% present. Meniscal injury was identified, and partial lateral meniscectomy, medial meniscus repair, and lateral meniscus repair were done in 1, 3, and 2 patients, respectively. Because of intercondylar notch impingement, notchplasty was performed in five patients (27.78%). The mean length of harvested PLT graft was 25.7±2.9 (23-30) cm while the mean diameter of the doubled graft was 8.1±0.54 (7.3-8.9) mm.

**Table I:** Demographic data and baseline characteristics.

S.N.	Demographic variables		N (%)
1	Age (year)1		30.1 ± 11.8 (23-47)
2	Sex	male	18 (100)
3	Side involved	right left	13 (72.22) 5 (27.78)
4	Body mass index, BMI (kg/m^2^)^1^		23 ± 2.73
5	Graft used in primary surgery	Hamstring autograft	15 (83.33)
		Bone patellar tendon bone autograft	3 (16.67)
6	Sport activity before primary injury	Hockey	2 (11.11)
		football	3 (16.67)
		kabaddi	10 (55.56)
		athlete	3 (16.67)
7	Time to primary ACLR to revision ACLR (months)^1^		17.3 ± 2.1 (14-20)
8	Traumatic Mechanism of graft failure		16 (88.89)
9	Cartilaginous injury at the time of presentation	patellar	3 (16.67)
		Lateral side	5 (27.78)
		Medial side	6 (33.33)
10	Status of medial meniscus injury	No injury	10 (55.56)
		Meniscectomy done	4 (22.22)
		Repair was done	1 (5.56)
		Tear present	3 (16.67)
11	Status of lateral meniscus injury	No injury	11 (61.11)
		Meniscectomy done	3 (16.67)
		Repair was done	1 (5.56)
		Tear present	3 (16.67)
12	Limb malalignment in coronal plane	Varus	1 (5.56)
		Valgus	1 (5.56)
13	Femoral tunnel malposition		9 (50)
14	Notchplasty		5 (27.78)
15	Harvested Peroneus longus graft^1^	Length (cm)	25.7 ± 2.9 (23-30)
		Doubled graft diameter (mm)	8.1 ± 0.54 (7.3-8.9)
16	Follow-up (months)^1^		21.7 ± 3.2 (18-30)

Note: ^1^Data is presented in Mean ± SD (Range)

There was significant improvement in Lachman test, Pivot shift test and Rolimeter side to side difference postoperatively (p<0.001) (Table II). At the 18-month follow-up, none of the patients exhibited any signs of anterior laxity. Lysholm score, IKDC subjective score, Tegner score, and VAS score improved significantly at 18 months of follow-up post-operatively (p<0.001) (Table III). No patient complained of any graft site morbidity. There was no significant difference in AOFAS score between both the sides. The AOFAS score did not show any significant difference between the two sides of the legs (p=0.38). According to the Marx Activity Rating Scale, the patient's level of sporting activity increased significantly at the 18 months following surgery as compared to pre-operatively (p<0.001).

**Table II:** Comparison of pre-operative and post-operative clinical variables after the revision ACLR.

S.N.			Pre-operative (N)	Post-operative (N)	p-value
1	Lachman test	Grade 1, (2-5mm)	1	2	<0.001
		Grade 2, (6-10mm)	10	1	
		Grade 3, (> 10mm)	7	0	
		absent	0	15	
2	Pivot shift test	Grade 1, (glide)	1	1	<0.001
		Grade 2, (clunk)	4	1	
		Grade 3, (subluxation)	13	0	
		absent	0	16	
3	Rolimeter side to side difference (mm)^1^		6.11 ± 2.6 (6-13)	1.8 ± 1.3 (0-6)	<0.001

Note: ^1^Data is presented in Mean ± SD (Range)

**Table III:** Comparison between pre-operative and functional outcomes at 18 months follow-up, after revision ACLR + LET.

S.N.	Functional score		Pre-operative [Mean ± SD]	At 18 months follow-up, after revision ACLR + LET [Mean ± SD]	p-value
1	Lysholm score		57.3±18.06	91.1 ± 7.9	<0.001
2	IKDC subjective score		59.5 ± 15.8	84.3 ± 13.2	<0.001
4	Tegner		4 ± 1.6	7 ± 1.9	<0.001
5	VAS		5.1 ± 1.8	2.7 ± 1.8	<0.001
6	MARX Activity Rating Scale (MARS)		8.12 ± 3.04	11.31 ± 3.21	<0.001
7	AOFAS	Donar side	99.1 ± 0.4	0.38	
		Opposite side	98.8 ± 1.4		

**Fig. 3:**
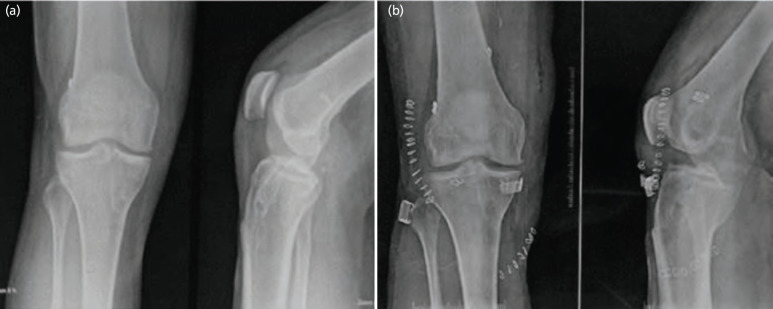
(a) Pre-operative radiograph of 26-year-old male, (b) post-operative radiograph of the same patient managed with revision ACLR + LET.

All the patients were satisfied with the outcome of the surgery. Six (33.33%) patients decided to partake in a less strenuous activity for personal and familial reasons, while 12 (66.67%) patients resumed their preinjury sport and physical activity levels. There was no infection or failure of surgery in any case (Table IV). The average follow-up time was 21.7±3.2 (18-30) months. Fig. 3 shows the pre-operative and post-operative radiographs of the right knee of a 26-year-old male patient with a failure of primary ACLR, managed by revision ACLR by PLT autograft with LET.

**Table IV:** Post-operative level of satisfaction and return to sport activity.

S.N.	Variable		Number, N (%)
1	Satisfy with the outcomes of surgery	Yes	18 (100)
2	Return to sport and physical activity	same	12 (66.67)
		lower	6 (33.33)
3	Failed revision ACLR		0
4	Complications		0

## Discussion

The major result of this study was that an additional LET in a single-stage revision ACL reconstruction using the PLT improved the outcome of patients with high-grade anterior laxity of knee before surgery with comparable graft donor site morbidity. Further, in this high-risk patient population, an additional LET can decrease failure rates and increase postoperative functional scores. To the best of our knowledge, only a few authors have documented single-stage revision ACLR using autologous PLT paired with LET.

On a technical level, revision ACLR surgery is more difficult and has a higher failure rate than original reconstruction^3,5,6^. Previous study has shown that patients having high-grade knee laxity might benefit from additional stabilisation surgeries because increasing laxity may be linked to injuries to the anterolateral complex of the knee^3,9,19^. Recent biomechanical studies have shown that LET can decrease the chance of failure by reducing the force acting on the ACL^7,10,11^.

Alm *et al*^13^ retrospectively studied 75 revision ACLR patients with primary ACLR graft failure and high-grade anterior instability of the knee, out of whom 59 revision ACLR patients had modified Lemaire tenodesis. They noticed that failure of the revision ACLR occurred in 8.2% of the cases and that, compared to patients without LET, patients with revision ACLR and high-grade anterior instability of knee had significantly improved functional outcomes, significantly lower failure rates, and a lower incidence of a positive pivot shift. They came to the conclusion that extra LET considerably lowers the chance of revision ACLR failure, the incidence of pivot-shift, and the post-operative functional result in patients with high-grade anterior instability and revision ACLR.

Keizer *et al*^34^ conducted a retrospective cohort study on 78 patients who underwent revision ACLR with an autologous ipsilateral bone-patellar tendon-bone autograft with and without LET, assessing their ability to return to the sports they played prior to the injury, their scores on the Knee Injury and Osteoarthritis Outcome Score, their answers to the subjective form of the International Knee Documentation Committee, and their Tegner activity score. They reported that 52% of patients who received revision ACLR with LET returned to the type of sport they were doing prior to their injury, compared to 31% of patients who underwent revision ACLR without LET (p=0.05). They came to the conclusion that an additional LET enhances the rate of pre-injury types of sports following revision ACLR since no significant differences were identified in the KOOS, IKDC, or Tegner activity scores.

One of the most significant factors to consider during ACL restoration surgery on the knee is the diameter of the graft. ACL reconstruction with quadrupled hamstring autograft with a diameter of 8mm or above reduces failure rates^35^. Another study demonstrated a strong positive link between a 1mm increase in graft diameter and a higher KOOS and IKDC score, as well as a greater revision rate for graft sizes smaller than 8mm^36^. One of the most important aspects of ACL reconstruction procedures is selecting the graft to be used. Compared to the quadrupled hamstring autograft and the quadriceps tendon and patellar tendon, the tensile strength of the PLT is analogous, according to biomechanical tests^37,38^.

In their comparative study, Rhatomy *et al*^23^, Agarwal *et al*^24^ and Keyhani *et al*^39^ reported that there were no significant differences between the pre-operative and post-operative scores between the hamstring and peroneus longus groups in the IKDC and Lysholm knee score, the AOFAS, FADI, and ankle ROM, with a significant improvement in thigh muscle wasting among the PLT group at final follow-up with the significantly larger diameter of the PLT graft than the hamstring graft.

In the review, He *et al*^40^ reported that, for ACL reconstruction, PLT autograft showed equivalent functional outcomes and graft survival rates to HT autograft; however, a minor decline in the AOFAS score should be taken into account during surgical planning. They also reported that, in order to prevent the quadriceps-hamstring imbalance that might happen when harvesting autografts from the knee, the PLT is an appropriate autograft obtained outside the knee for ACL reconstruction.

However, there are very few studies showing the results of revision ACL reconstruction using PLT. In a prospective study Goyal *et al*^41^ reported significant improvement in the VAS scores for pain, Lysholm, and IKDC at two years follow-up. They also reported nonsignificant donor site morbidity in terms of AOFAS score, first ray plantarflexion strength, ankle dorsiflexion, ankle plantarflexion, and eversion strength, which were comparable to the normal side, and reported no incidences of transplant failure, superficial infection, or deep infection.

In the present study, we found the length of PLT to be 25.7±2.9 (23–30) cm and the diameter of the doubled graft to be 8.1±0.54 (7.3–8.9) mm. These findings were comparable with previous studies^24,41^. Our study showed significant improvement in the results of the IKDC score, Tegner score, and Lysholm score. After harvesting the PLT, the donor's ankle function was still good according to the AOFAS score. This is probable because the donor ankle has a healthy peroneus brevis, which replaced ankle eversion after PLT harvesting.

In our study, it showed that the activity level at the 18-month follow-up was very similar to the pre-injury level and that there was no incidence of graft failure at later follow-ups. In a retrospective study Zanna *et al*^42^ found that 58.8% of the patients returned to their desired level of sport after revision ACLR using BPTBG with LET. Alessio-Mazzola *et al*^43^ reported that 91.7% of the Professional Soccer Players were returned to the same level of sport after revision ACLR using bone-patellar tendon-bone autograft or a hamstring graft with LET. In the present study, all the patients were satisfied with the surgical outcome, out of which 12 (66.67%) returned to their preinjury sport and physical activity level.

The survival and function of the graft should be monitored for a longer period of time, preferably with randomisation and a control group receiving different grafts. A further limitation of the current study is that it is centred on a small sample size of patients undergoing revision ACLR. The dependability of the observer was also crucial to the clinical evaluation. Long-term problems such as osteoarthritis after surgery could not be evaluated due to the short follow-up period.

## Conclusions

The rate of return to the pre-injury type of physical activity and sports at 18 month follow-up is increased in patients who receive a combined LET and revision ACLR using PLT which is an appropriate autograft option after a primary ACLR failure without significate donor site morbidity. Additional LET is a safe procedure and considerably lowers the likelihood of revision ACLR failure and the incidence of pivot shift among those with revision ACLR and high-grade anterior instability, while simultaneously improving postoperative functional results.

## References

[1] Lien-Iversen T, Morgan DB, Jensen C, Risberg MA, Engebretsen L, Viberg B (2020). Does surgery reduce knee osteoarthritis, meniscal injury and subsequent complications compared with non-surgery after ACL rupture with at least 10 years follow-up? A systematic review and meta-analysis.. Br J Sports Med..

[2] Grassi A, Kim C, Marcheggiani Muccioli GM, Zaffagnini S, Amendola A (2017). What Is the Mid-term Failure Rate of Revision ACL Reconstruction? A Systematic Review.. Clin Orthop Relat Res..

[3] Ferretti A, Monaco E, Ponzo A, Basiglini L, Iorio R, Caperna L (2016). Combined Intra-articular and Extra-articular Reconstruction in Anterior Cruciate Ligament-Deficient Knee: 25 Years Later.. Arthroscopy..

[4] Kocher MS, Steadman JR, Briggs K, Zurakowski D, Sterett WI, Hawkins RJ (2002). Determinants of patient satisfaction with outcome after anterior cruciate ligament reconstruction.. J Bone Joint Surg Am..

[5] Magnussen RA, Reinke EK, Huston LJ, MOON Group, Hewett TE, Spindler KP (2016). Effect of High-Grade Preoperative Knee Laxity on Anterior Cruciate Ligament Reconstruction Outcomes.. Am J Sports Med..

[6] Sonnery-Cottet B, Thaunat M, Freychet B, Pupim BH, Murphy CG, Claes S (2015). Outcome of a Combined Anterior Cruciate Ligament and Anterolateral Ligament Reconstruction Technique With a Minimum 2-Year Follow-up.. Am J Sports Med..

[7] Getgood AMJ, Bryant DM, Litchfield R, Heard M, McCormack RG, Rezansoff A (2020). Lateral Extra-articular Tenodesis Reduces Failure of Hamstring Tendon Autograft Anterior Cruciate Ligament Reconstruction: 2-Year Outcomes From the STABILITY Study Randomized Clinical Trial.. Am J Sports Med..

[8] Claes S, Vereecke E, Maes M, Victor J, Verdonk P, Bellemans J (2013). Anatomy of the anterolateral ligament of the knee.. J Anat..

[9] Sonnery-Cottet B, Lutz C, Daggett M, Dalmay F, Freychet B, Niglis L (2016). The Involvement of the Anterolateral Ligament in Rotational Control of the Knee.. Am J Sports Med..

[10] Devitt BM, Neri T, Fritsch BA (2023). Combined anterolateral complex and anterior cruciate ligament injury: Anatomy, biomechanics, and management-State-of-the-art.. J ISAKOS..

[11] Porter MD, Shadbolt B, Pomroy S (2018). The Augmentation of Revision Anterior Cruciate Ligament Reconstruction With Modified Iliotibial Band Tenodesis to Correct the Pivot Shift: A Computer Navigation Study.. Am J Sports Med..

[12] Redler A, Iorio R, Monaco E, Puglia F, Wolf MR, Mazza D (2018). Revision Anterior Cruciate Ligament Reconstruction With Hamstrings and Extra-articular Tenodesis: A Mid- to Long-Term Clinical and Radiological Study.. Arthroscopy..

[13] Alm L, Drenck TC, Frosch KH, Akoto R (2020). Lateral extra-articular tenodesis in patients with revision anterior cruciate ligament (ACL) reconstruction and high-grade anterior knee instability.. Knee..

[14] Eggeling L, Drenck TC, Frings J, Krause M, Korthaus A, Krukenberg A (2022). Additional lateral extra-articular tenodesis in revision ACL reconstruction does not influence the outcome of patients with low-grade anterior knee laxity.. Arch Orthop Trauma Surg..

[15] Cantin O, Lustig S, Rongieras F, Saragaglia D, Lefèvre N, Graveleau N (2016). Outcome of cartilage at 12years of follow-up after anterior cruciate ligament reconstruction.. Orthop Traumatol Surg Res..

[16] Imbert P, Lustig S, Steltzlen C, Batailler C, Colombet P, Dalmay F (2017). Midterm results of combined intra- and extra-articular ACL reconstruction compared to historical ACL reconstruction data. Multicenter study of the French Arthroscopy Society.. Orthop Traumatol Surg Res..

[17] Spindler KP, Huston LJ, Chagin KM, Kattan MW, Reinke EK (2018). Ten-Year Outcomes and Risk Factors After Anterior Cruciate Ligament Reconstruction: A MOON Longitudinal Prospective Cohort Study.. Am J Sports Med..

[18] Nitri M, Rasmussen MT, Williams BT, Moulton SG, Cruz RS, Dornan GJ (2016). An In Vitro Robotic Assessment of the Anterolateral Ligament, Part 2: Anterolateral Ligament Reconstruction Combined With Anterior Cruciate Ligament Reconstruction.. Am J Sports Med..

[19] Williams A, Ball S, Stephen J, White N, Jones M, Amis A (2017). The scientific rationale for lateral tenodesis augmentation of intra-articular ACL reconstruction using a modified 'Lemaire' procedure.. Knee Surg Sports Traumatol Arthrosc..

[20] Park JG, Han SB, Lee CS, Jeon OH, Jang KM (2022). Anatomy, Biomechanics, and Reconstruction of the Anterolateral Ligament of the Knee Joint.. Medicina (Kaunas)..

[21] Mayr R, Sigloch M, Coppola C, Hoermann R, Iltchev A, Schmoelz W (2022). Modified Lemaire tenodesis reduces anterior cruciate ligament graft forces during internal tibial torque loading.. J Exp Orthop..

[22] Inderhaug E, Stephen JM, Williams A, Amis AA (2017). Biomechanical Comparison of Anterolateral Procedures Combined With Anterior Cruciate Ligament Reconstruction.. Am J Sports Med..

[23] Rhatomy S, Asikin AIZ, Wardani AE, Rukmoyo T, Lumban-Gaol I, Budhiparama NC (2019). Peroneus longus autograft can be recommended as a superior graft to hamstring tendon in single-bundle ACL reconstruction.. Knee Surg Sports Traumatol Arthrosc..

[24] Agarwal A, Singh S, Singh A, Tewari P (2023). Comparison of Functional Outcomes of an Anterior Cruciate Ligament (ACL) Reconstruction Using a Peroneus Longus Graft as an Alternative to the Hamstring Tendon Graft.. Cureus..

[25] Shi FD, Hess DE, Zuo JZ, Liu SJ, Wang XC, Zhang Y (2019). Peroneus Longus Tendon Autograft is a Safe and Effective Alternative for Anterior Cruciate Ligament Reconstruction.. J Knee Surg..

[26] Singh H, McKay M, Baldwin J, Nicholson L, Chan C, Burns J (2017). Beighton scores and cut-offs across the lifespan: cross-sectional study of an Australian population.. Rheumatology (Oxford)..

[27] Risberg MA, Holm I, Steen H, Beynnon BD (1999). Sensitivity to changes over time for the IKDC form, the Lysholm score, and the Cincinnati knee score. A prospective study of 120 ACL reconstructed patients with a 2-year follow-up.. Knee Surg Sports Traumatol Arthrosc..

[28] Tegner Y, Lysholm J, Odensten M, Gillquist J (1988). Evaluation of cruciate ligament injuries. A review.. Acta Orthop Scand..

[29] Irrgang JJ, Anderson AF (2002). Development and validation of health-related quality of life measures for the knee.. Clin Orthop Relat Res..

[30] Collins NJ, Prinsen CA, Christensen R, Bartels EM, Terwee CB, Roos EM (2016). Knee Injury and Osteoarthritis Outcome Score (KOOS): systematic review and meta-analysis of measurement properties.. Osteoarthritis Cartilage..

[31] Ohnhaus EE, Adler R (1975). Methodological problems in the measurement of pain: a comparison between the verbal rating scale and the visual analogue scale.. Pain..

[32] Marx RG, Stump TJ, Jones EC, Wickiewicz TL, Warren RF (2001). Development and evaluation of an activity rating scale for disorders of the knee.. Am J Sports Med..

[33] Van Lieshout EM, De Boer AS, Meuffels DE, Den Hoed PT, Van der Vlies CH, Tuinebreijer WE (2017). American Orthopaedic Foot and Ankle Society (AOFAS) Ankle-Hindfoot Score: a study protocol for the translation and validation of the Dutch language version.. BMJ Open..

[34] Keizer MNJ, Brouwer RW, de Graaff F, Hoogeslag RAG (2023). Higher return to pre-injury type of sports after revision anterior ligament reconstruction with lateral extra-articular tenodesis compared to without lateral extra-articular tenodesis.. Knee Surg Sports Traumatol Arthrosc..

[35] Conte EJ, Hyatt AE, Gatt CJ Jr, Dhawan A (2014). Hamstring autograft size can be predicted and is a potential risk factor for anterior cruciate ligament reconstruction failure.. Arthroscopy..

[36] Mariscalco MW, Flanigan DC, Mitchell J, Pedroza AD, Jones MH, Andrish JT (2013). The influence of hamstring autograft size on patient-reported outcomes and risk of revision after anterior cruciate ligament reconstruction: a Multicenter Orthopaedic Outcomes Network (MOON) Cohort Study.. Arthroscopy..

[37] Hossain GMJ, Islam MS, Rahman Khan MM, Rafiqul Islam M, Rahman SMM, Jahan MS (2023). A prospective study of arthroscopic primary ACL reconstruction with ipsilateral peroneus longus tendon graft: Experience of 439 cases.. Medicine (Baltimore)..

[38] Malige A, Baghdadi S, Hast MW, Schmidt EC, Shea KG, Ganley TJ (2022). Biomechanical properties of common graft choices for anterior cruciate ligament reconstruction: A systematic review.. Clin Biomech (Bristol, Avon)..

[39] Keyhani S, Qoreishi M, Mousavi M, Ronaghi H, Soleymanha M (2022). Peroneus Longus Tendon Autograft versus Hamstring Tendon Autograft in Anterior Cruciate Ligament Reconstruction: A Comparative Study with a Mean Follow-up of Two Years.. Arch Bone Jt Surg..

[40] He J, Tang Q, Ernst S, Linde MA, Smolinski P, Wu S (2021). Peroneus longus tendon autograft has functional outcomes comparable to hamstring tendon autograft for anterior cruciate ligament reconstruction: a systematic review and meta-analysis.. Knee Surg Sports Traumatol Arthrosc..

[41] Goyal T, Paul S, Choudhury AK, Sethy SS (2023). Full-thickness peroneus longus tendon autograft for anterior cruciate reconstruction in multi-ligament injury and revision cases: outcomes and donor site morbidity.. Eur J Orthop Surg Traumatol..

[42] Zanna L, Niccolò G, Matteo I, Malone J, Roberto C, Fabrizio M (2023). Clinical outcomes and return to sport after single-stage revision anterior cruciate ligament reconstruction by bone-patellar tendon autograft combined with lateral extra-articular tenodesis.. Eur J Orthop Surg Traumatol..

[43] Alessio-Mazzola M, Formica M, Russo A, Sanguineti F, Capello AG, Lovisolo S, Felli L (2019). Outcome after Combined Lateral Extra-articular Tenodesis and Anterior Cruciate Ligament Revision in Professional Soccer Players.. J Knee Surg..

